# A robust cooperative localization algorithm based on covariance intersection method for multi-robot systems

**DOI:** 10.7717/peerj-cs.1373

**Published:** 2023-05-12

**Authors:** Miao Wang, Qingshan Liu

**Affiliations:** 1 School of Cyber Science and Engineering, Southeast University, Nanjing, China; 2 Purple Mountain Laboratories, Nanjing, China; 3 School of Mathematics, Southeast University, Nanjing, China

**Keywords:** Cooperative localization, Robust localization, Multi-robot system, Covariance intersection, Kullback–Leibler divergence

## Abstract

Cooperative localization is an arising research problem for multi-robot system, especially for the scenarios that need to reduce the communication load of base stations. This article proposes a novel cooperative localization algorithm, which can achieve high accuracy localization by using the relative measurements among robots. To address uncertainty in the measuring robots’ positions and avoid linearization errors in the extended Kalman filter during the measurement update phase, a particle-based approximation method is proposed. The covariance intersection method is then employed to fuse preliminary estimations from different robots, guaranteeing a minimum upper bound for the fused covariance. Moreover, in order to avoid the negative effect of abnormal measurements, this article adopts the Kullback–Leibler divergence to calculate the distances between different estimations and rejects to fuse the preliminary estimations far from the estimation obtained in the prediction stage. Two simulations are conducted to validate the proposed algorithm. Compared with the other three algorithms, the proposed algorithm can achieve higher localization accuracy and deal with the abnormal measurement.

## Introduction

Localization is a fundamental requirement in many engineering applications (Bi et al., 2021; Poursheikhali & Zamiri-Jafarian, 2021), such as surveillance (Ferri et al., 2017), environmental monitoring (Mesmoudi, Feham & Labraoui, 2013), target tracking (Morbidi & Mariottini, 2012) and intelligent navigation (Lee, 2021). With the progress of localization technology and positioning accuracy requirements, cooperative localization is becoming a promising localization method under complicated environments (Wymeersch, Lien & Win, 2009; Van Nguyen et al., 2015; Huang et al., 2017; Fathy et al., 2021). Different from traditional localization algorithms, cooperative localization algorithm emphasizes the relative measurements between robots and the inter-robot communication during the localization process (Zhu, 2020). It is also the reason that the cooperative localization algorithm is mainly used in multi-robot system. Moreover, cooperative localization algorithm has higher accuracy in complicated environment since robots can jointly process the relative measurements. Another advantage of the cooperative localization is that it doesn’t require to place other redundant sensors in environment, which leads lower cost than traditional localization methods.

In fact, there are many scientists who study cooperative localization (Kurazume, Nagata & Hirose, 1994; Patwari et al., 2005; Van Nguyen et al., 2015; Huang et al., 2021). According to the different framework adopted, the existing research can be divided into two categories: filter-based and optimization-based methods (Chang, Chen & Mehta, 2021; Garcia-Fernandez, Svensson & Särkkä, 2017; Prorok, Bahr & Martinoli, 2012). Among them, the filter-based method includes extended Kalman filter and particles filter (Indelman et al., 2012; Fox et al., 2000). For optimization-based method, it includes maximum likelihood estimation (Howard, Matarić & Sukhatme, 2003) and maximum posteriori estimation (Nerurkar, Roumeliotis & Martinelli, 2009; Liu, Lian & Zhou, 2019). Comparing the filter-based method, the optimization-based method requires excessive internal communication and lots of complicated calculation (Chang, Chen & Mehta, 2021), which is also the reason that this article mainly focuses on the filter-based method.

The filter-based methods are the common method for cooperative localization of multi-robot system. In Xu, Asghar & Liu (2019), a Huber-based robust algorithm is proposed for cooperative localization of autonomous underwater vehicles, which adopts an adaptive noise estimation method to estimate the non-Gaussian noise. In Fang, Li & Yang (2021), an adaptive cubature split covariance intersection filter is used to estimate the vehicle state, the advantage of which is that it uses the cubature transform and innovation-based adaptive estimation theory to deal with dynamic measurement noise. However, these two works did not consider the scenes with abnormal measurements. In order to handle the spurious sensor data, Wang et al. (2021) presents a cooperative localization algorithm based on covariance union (CU) that simply uses the properties of the CU method to obtain a consistent estimate. However, the method can only handle the spurious sensor data with small deviation, but not those with large deviation in malicious attack or non-line-of-sight (NLOS) scenarios. Moreover, in Chang, Chen & Mehta (2020), a cooperative localization method based on covariance intersection (CI) is proposed and verified in experiments, which has the same problem of not being able to process the spurious sensor data with large deviations. Based on the above research, this article tries to study the cooperative localization problem and improves the robustness of the cooperative localization algorithm for large abnormal measurements.

In this article, a novel cooperative localization algorithm based on CI is proposed to locate robots in multi-robot system. A robot can achieve high accuracy localization by fusing the preliminary estimations from its neighbour robots. To improve the robustness of the localization algorithm, a particle-based approximation method is proposed to avoid the errors caused by linearization when accounting for the uncertainty in the positions of the neighbours. Furthermore, the Kullback–Leibler divergence (KLD) is used to handle the case with abnormal measurements. The main contributions of this article can be summarized as follows.

(1) An approximation method based on particles is proposed to account for the uncertainty of the positions of robots. This process can improve the accuracy of the extended Kalman filter in positioning.
(2) A cooperative localization algorithm based on the CI method is proposed to achieve localization by fusing the preliminary estimations. And the advantage of this algorithm is that it can achieve high localization accuracy by minimizing the trace of the fused estimation covariance.
(3) The KLD theory is used to eliminate the abnormal measurement and provides a robust estimation for robot.

## Preliminary work

In this section, both the kinematic and measurement model of the robot used are presented firstly. Then an approximate method based on particles is proposed to adjust the measurement noise for considering the uncertainty of robots’ positions.

### Kinematic model of robot

Generally, a robot’s kinematic model depends on its type and the modeling method. In this article, robots are assumed with nonholonomic constraint and only permitted to move on the horizontal plane. So the position and orientation of robots are only needed to consider that on the plane, which correspond to two and one dimension respectively. Moreover, the control variables of the robot only include linear velocity $v$ and angular velocity $\omega$. Supposing the state of robot at time $k$ is ${X_k} = {[{q_k},{\theta _k}]^ \top }$, where ${q_k} = [{x_k},{y_k}]$ denotes the position of the robot and ${\theta _k}$ is the orientation angle. Then the kinematic model of the robot can be presented as

(1) $${X_{k + 1}} = f({X_k},{u_k},{\delta _k}) = \left[ {\matrix{ {{x_k} + ({v_k} + {\delta _v})T\cos ({\theta _k})} \cr  {{y_k} + ({v_k} + {\delta _v})T\sin ({\theta _k})} \cr  {{\theta _k} + ({\omega _k} + {\delta _\omega })T} \cr  } } \right]$$

where $f( \cdot )$ is the nonlinear state propagation function of the robot, ${u_k} = {[{v_k},{\omega _k}]^ \top }$ is the control input at time $k$, *T* is the sampling time interval, and ${\delta _k} = {\rm{diag}}\left( {[{\delta _v},{\delta _\omega }]} \right)$ is the input noise obeying a zero-mean Gaussian distribution with variance *Q*.

### Measurement model

The measurement model commonly depends on the type of sensors used. In this work, the measurement information only considers the distance between robots, which can be obtained by the range sensing sensors, such as ultra-wide bandwidth (UWB) device and Zigbee. So the measurement model can be represented as

(2) $${z_{ij}} = h({q_i},{q_j}) = ||{q_i} - {q_j}|| + {\eta _{ij}}$$

where $h( \cdot )$ is the measurement function, $|| \cdot ||$ is the Euclidean norm, ${q_i}$ and ${q_j}$ are the positions of robots $i$ and $j$. Moreover, ${\eta _{ij}}$ is the measurement noise obeying a zero-mean Gaussian distribution with covariance *R*.

### Method for considering measurement uncertainty

The above measurement model is based on the assumption that both the two robots’ positions are accurate. However, in real application, robots’ position are commonly a series of estimations with variance, that is, their position are inaccurate. In the cooperative localization applications, if we directly adopt the measured distance between two robots without considering the uncertainty of its position, it might enlarge the variance of the fused estimation and cause the cooperative localization algorithm to diverge. Conversely, if we consider the uncertainty of robots’ positions and select the measurements from those robots with low position uncertainty to conduct the fusion estimation, the positioning accuracy of the cooperative localization algorithm can certainly be improved. In order to consider the uncertainty of robots’ positions, an immediate idea is adjusting the measurement noise by using its marginal distribution after combine the uncertainty of robots’ positions and the measurement noise as a joint probability distribution.

As described in Eq. (2), the measurement noise is assumed as ${\eta _{ij}}\sim{\cal N}(0,R)$. For the measured distance ${z_{ij}}$, it can also be considered that it obeys the same distribution, so its probability density function can be represented as

(3) $$p({z_{ij}}\mid {x_i},{x_j}) = {1 \over {\sqrt {2\pi } R}}{\rm{exp}}\left\{ {{{{{\left( {{z_{ij}} - ||{x_i} - {x_j}||} \right)}^2}} \over {2{R^2}}}} \right\}$$

where the conditional probability $p({z_{ij}}\mid {x_i},{x_j})$ means that the measured distance ${z_{ij}}$ is correlated with the positions of ${x_i}$ and ${x_j}$. In real scenarios, if the position robots are known and certain, their probability distribution can be considered as the delta distribution. Then the joint probability can be represented as

(4) $$p({z_{ij}},{x_i},{x_j}) = p({x_i})p({x_j})p({z_{ij}}\mid {x_i},{x_j})$$

So the measurement with considering the uncertainty of robots’ positions is obeyed to

(5) $$\eqalign {p({z_{ij}})& = \int\!\!\!\int p ({x_i})p({x_j})p({z_{ij}}\mid {x_i},{x_j})d{x_i}d{x_j}\\ &= \int\!\!\!\int \delta ({x_i} - {\mu _i})\delta ({x_j} - {\mu _j}){1 \over {\sqrt {2\pi } R}}{\rm{exp}}\left\{ {{{{{\left( {{z_{ij}} - ||{x_i} - {x_j}||} \right)}^2}} \over {2{R^2}}}} \right\}d{x_i}d{x_j}\\ &= {1 \over {\sqrt {2\pi } R}}{\rm{exp}}\left\{ {{{{{\left( {{z_{ij}} - ||{\mu _i} - {\mu _j}||} \right)}^2}} \over {2{R^2}}}} \right\}\\ &= {\cal N}(||{\mu _i} - {\mu _j}||,R)}$$

where $\delta ( \cdot )$ denotes the delta distribution, ${\mu _i}$ and ${\mu _j}$ are the real positions of robots $i$ and $j$, respectively. The third equality of Eq. (5) is obtained by using the characteristic of delta distribution, and Eq. (5) denotes the marginal probability of the measurement has a same distribution to the measurement noise when robots’ positions are accurate.

**Figure 1:**
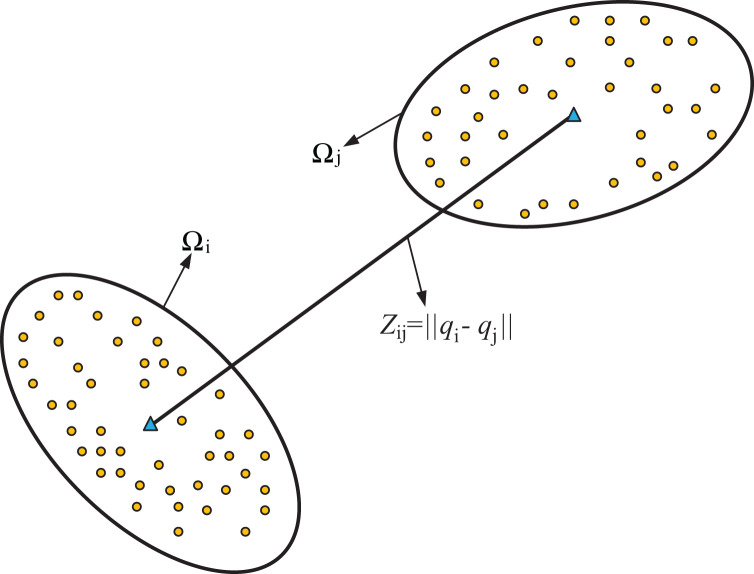
Illustration of the method for accounting for the uncertainty of measurement. ${\Omega _i}$ and ${\Omega _j}$ are the probability distribution regions of robot positions ${q_i}$ and ${q_j}$ respectively.

It is worth noting that the conclusion in Eq. (5) cannot be obtained if the positions of the robots are inaccurate. Additionally, calculating the detailed marginal distribution can be challenging due to the nonlinear mapping of the term $p({z_{ij}}\mid {x_i},{x_j})$. To address this issue, a common approach is to linearize the measurement model Eq. (2), which is typically used in the extended Kalman filter. However, when considering the uncertainty of both robots, linearizing the Eq. (2) model at the positions of the two robots can introduce linearization errors twice. This can reduce the estimation accuracy of the extended Kalman filter during the positioning process. To address these challenges and avoid linearization errors, an approximation method based on particles has been proposed. This approach enables the consideration of uncertainty of robots and can be used in the extended Kalman filter without introducing the two linearization errors. The basic idea of the method can be viewed in Fig. 1. As shown in Fig. 1, measurement *Z* is the measured distance between robots ${p_1}$ and ${p_2}$. Two groups of particles are generated according to the probability distribution of robots’ positions. Then the method calculates the distances between particles from different groups and obtains the variance between the distances and the measured distance *Z*. The details of the method are presented in Algorithm 1.

**Algorithm 1:** An approximate method for accounting for the uncertainty of measurement

**Initialization:**
1: Input the probability distributions of robots’ positions $P({q_i})$ and $P({q_j})$, the measurement distance *z*_*ij*_, the covariance *R* of the measurement noise ${\eta _{ij}}$ and the particles number *L*.
**Iteration:**
2: Based on the positions’ distributions $P({q_i})$ and $P({q_j})$, using the sampling method to generate two particle groups with *L* particles, ${\Omega _{{q_i}}}$ and ${\Omega _{{q_j}}}$.
3: Calculate the distances between all two particles from different groups and denote the result as $D = \left\{ {||s - a||\mid s \in {\Omega _{{q_i}}},a \in {\Omega _{{q_j}}}} \right\}$.
4: Minus the measurement distance *z*_*ij*_ from every distance in *D* to obtain the deviation set $C = \left\{ {{z_{ij}} - u\mid u \in D} \right\}$
5: Add the variance of deviation set *C* the covariance *R* to obtain the uncertainty value *S*.
6: **return** S

## Cooperative localization

In this section, a novel cooperative localization based CI method is proposed to fuse preliminary estimations. And the KLD theory is used to detect the abnormal measurement and improve the robustness of the cooperative localization algorithm.

### State prediction process

Similar to the extended Kalman filter, the novel cooperative localization method consists of two parts, namely the state prediction process and the measurement update process. Besides, the two method have a same state prediction process, and their difference is in the measurement update process. In order to fit the theme of the cooperative localization, here we consider a multi-robot system with N robots, which can be denoted as $\{ {R_1},{R_2}, \ldots ,{R_N}\}$. If we distinguish robot’s state in two process by using different subscripts, then the predicted state of robot ${R_i}$ at time $k$ can be denoted as $X_{k + 1|k}^{{R_i}}$, and the updated state of robot ${R_i}$ at time $k$ is $X_k^{{R_i}}$. Correspondingly, the state prediction equation of robot can be represented as

(6) $$X_{k + 1|k}^{{R_i}} = A_k^{{R_i}}X_k^{{R_i}} + B_k^{{R_i}}u_k^{{R_i}}$$

where $A_k^{{R_i}}$ is the Jacobian matrix of Eq. (1) with respect to $X_k^{{R_i}}$, and $B_k^{{R_i}}$ is the Jacobian matrix of Eq. (1) with respect to $u_k^{{R_i}}$. The two Jacobian matrices can be calculated according to

(7) $$A_k^{{R_i}} = \left[ {\matrix{ 1 & 0 & { - v_k^{{R_i}}T\sin (\theta _k^{{R_i}})} \cr  0 & 1 & {v_k^{{R_i}}T\cos (\theta _k^{{R_i}})} \cr  0 & 0 & 1 \cr  } } \right]\quad B_k^{{R_i}} = \left[ {\matrix{ {T\cos (\theta _k^{{R_i}})} & 0 \cr  {T\sin (\theta _k^{{R_i}})} & 0 \cr  0 & T \cr  } } \right]$$

Correspondingly, the covariance of robot ${R_i}$’s state is propagated as

(8) $$P_{k + 1\mid k}^{{R_i}} = A_k^{{R_i}}P_k^{{R_i}}{(A_k^{{R_i}})^ \top } + B_k^{{R_i}}Q{(B_k^{{R_i}})^ \top }$$

where ${P_k}$ is robot ${R_i}$’s state covariance at time $k$, and *Q* is the noise of input variable.

### Measurement update process

Based on the characteristics of the ranging sensor, robots ${R_i}$ and ${R_j}$ need to communicate with each other if a relative distance $Z_{k + 1}^{{R_i},{R_j}}$ between the two robots is measured. Through this communication, robots can also exchange their own state information and covariance. Supposing robot ${R_i}$ obtains $X_{k + 1\mid k}^{{R_j}}$ and $P_{k + 1\mid k}^{{R_j}}$ from robot ${R_j}$, then it can calculate a preliminary estimation based on the measurement $Z_{k + 1}^{{R_i},{R_j}}$. And the calculation of the preliminary estimation is similar to the process of the extended Kalman filter, which can be represented as

(9) $$X_{k + 1}^{{R_i},{R_j}} = X_{k + 1|k}^{{R_i}} + K_{k + 1}^{{R_i},{R_j}}(Z_{k + 1}^{{R_i},{R_j}} - h(X_{k + 1|k}^{{R_i}},X_{k + 1|k}^{{R_j}}))$$

(10) $$P_{k + 1}^{{R_i},{R_j}} = P_{k + 1|k}^{{R_i}} - K_{k + 1}^{{R_i},{R_j}}S_{k + 1}^{{R_i},{R_j}}{\left( {K_{k + 1}^{{R_i},{R_j}}} \right)^ \top }$$

(11) $$K_{k + 1}^{{R_i},{R_j}} = P_{k + 1|k}^{{R_i}}H_{k + 1}^{{R_i}}{(S_{k + 1}^{{R_i},{R_j}})^{ - 1}}$$

(12) $$S_{k + 1}^{{R_i},{R_j}} = H_{k + 1}^{{R_i}}P_{k + 1|k}^{{R_i}}{(H_{k + 1}^{{R_i}})^ \top } + R_{k + 1}^{{R_i},{R_j}}$$

where $H_{k + 1}^{{R_i}}$ is the Jacobian matrix of the measurement model with $X_{k + 1}^{{R_i},{R_j}}$, $R_{k + 1}^{{R_i},{R_j}}$ is the approximated measurement noise calculated by Algorithm 1.

**Remark 1.** *Actually, the calculation method of* $R_{k + 1}^{{R_i},{R_j}}$ *is the main difference between Eq. (12) and the extended Kalman filter without considering robots’ uncertainty. And in Eq. (12), the calculation of* $R_{k + 1}^{{R_i},{R_j}}$ *have considered the uncertainty of robots’ positions. Moreover, the first term of Eq. (12) has considered the uncertainly of robot* ${R_i}$*’s position, so it’s only need to consider the uncertainly of robot* ${R_j}$*’s position when calculating the value of* $R_{k + 1}^{{R_i},{R_j}}$. *In other words, it is only need to generate a group particles in application to approximate the probability distribution of* ${R_j}$*’s position, which is also a method to reduce the computation load*.

According to the description above, robot ${R_i}$ can obtain a preliminary estimation $(X_{k + 1}^{{R_i},{R_j}},P_{k + 1}^{{R_i},{R_j}})$ based on the measurement to robot ${R_j}$. If robot ${R_i}$ can communicate with multiple robots at the same time, it will have multiple preliminary estimations. Supposing robot ${R_i}$ has $m$ communication robots at time $k + 1$, then all preliminary estimations for robot ${R_i}$ can be denoted as $\{ (X_{k + 1}^{{R_i},{R_1}},P_{k + 1}^{{R_i},{R_1}}),(X_{k + 1}^{{R_i},{R_2}},P_{k + 1}^{{R_i},{R_2}}), \ldots ,$ $(X_{k + 1}^{{R_i},{R_m}},P_{k + 1}^{{R_i},{R_m}})\}$. In order to obtain the accurate estimation of robot ${R_i}$, the CI method can be used to fuse all preliminary estimations to generate the final fused estimation.

As an effective method fusing state estimations for the distributed system, CI method can provide a conservative estimation for robot ${R_i}$ based on its preliminary estimations (Chen, Arambel & Mehra, 2002; Arambel, Rago & Mehra, 2001; Carrillo-Arce et al., 2013). And the main formulas of the CI method can be represented as

(13) $$X_{k + 1}^{{R_i}} = P_{k + 1}^{{R_i}}\left[ {{\omega _1}{{\left( {P_{k + 1}^{{R_i},{R_1}}} \right)}^{ - 1}}X_{k + 1}^{{R_i},{R_1}} + \ldots + {\omega _{|{N_i}|}}{{\left( {P_{k + 1}^{{R_i},{R_{|{N_i}|}}}} \right)}^{ - 1}}X_{k + 1}^{{R_i},{R_{|{N_i}|}}}} \right]$$

(14) $${\left( {P_{k + 1}^{{R_i}}} \right)^{ - 1}} = {\omega _1}{\left( {P_{k + 1}^{{R_i},{R_1}}} \right)^{ - 1}} + \ldots + {\omega _{|{N_i}|}}{\left( {P_{k + 1}^{{R_i},{R_{|{N_i}|}}}} \right)^{ - 1}}$$

where ${\omega _s}$ is the weight of preliminary estimation $(X_{k + 1}^{{R_i},{R_s}},P_{k + 1}^{{R_i},{R_s}})$. Moreover, weight ${\omega _s}$ should meet the constraints with ${\omega _s} \in [0,1]$ and $\sum\nolimits_{s = 1}^{|{N_i}|} {{\omega _s}} = 1$. As for the selection of the optimal weight ${\omega _s}$, it is determined by minimizing an optimization problem such that

(15) $${\rm{min}}\;{\rm{tr}}(P_{k + 1}^{{R_i}}) = {\rm{min}}\;{\rm{tr}}\left\{ {{{\left[ {\sum\limits_{s = 1}^{|{N_i}|} {{\omega _s}} {{\left( {P_{k + 1}^{{R_i},{R_s}}} \right)}^{ - 1}}} \right]}^{ - 1}}} \right\}$$

where ${\rm{tr}}( \cdot )$ denotes the trace of the covariance matrix.

Obviously, the optimization problem in Eq. (15) is complex and has lot of computation, because it involves a large number of matrix inversion and optimization iteration. To handle this problem, a simplified method is used to optimize Eq. (15), which cancels the matrix inversion operation and directly uses the trace of the covariance matrix in the optimization. The simplified method is actually a generalization form of CI method, whose rationality and performance are validated in Daass, Pomorski & Haddadi (2021). For ease of understanding, here presents the core formula of the simplified method, which can be represented as

(16) $$\eqalign {{w_1} + {w_2} + \ldots + {w_{|{N_i}|}} &= 1 \\{w_{s - 1}}{\rm{tr}}(P_{k + 1}^{{R_i},{R_{s - 1}}}) - {w_s}{\rm{tr}}(P_{k + 1}^{{R_i},{R_s}})& = 0\quad (s = 2, \ldots ,|{N_i}|)}$$

If we write method Eq. (16) into compact matrix format, a set of equations can be obtained

(17) $$\left[ {\matrix{ {{\xi _1}} & { - {\xi _2}} & 0 & \ldots & 0 \cr 0 & {{\xi _2}} & { - {\xi _3}} & \ldots & 0 \cr \vdots & \vdots & \vdots & \ddots & \vdots \cr 0 & \ldots & 0 & {{\xi _{s - 1}}} & { - {\xi _s}} \cr 1 & \ldots & 1 & 1 & 1 \cr } } \right]\left[ {\matrix{ {{w_1}} \cr {{w_2}} \cr \vdots \cr {{w_{s - 1}}} \cr {{w_s}} \cr } } \right] = \left[ {\matrix{ 0 \cr 0 \cr \vdots \cr 0 \cr 1 \cr } } \right]$$

where ${\xi _s}$ is a shorthand for ${\rm{tr}}(P_{k + 1}^{{R_i},{R_s}})$, utilized to simplify the corresponding term in the matrix. It’s clearly that the computation load of Eq. (17) is far smaller than that of Eq. (15).

**Figure 2:**
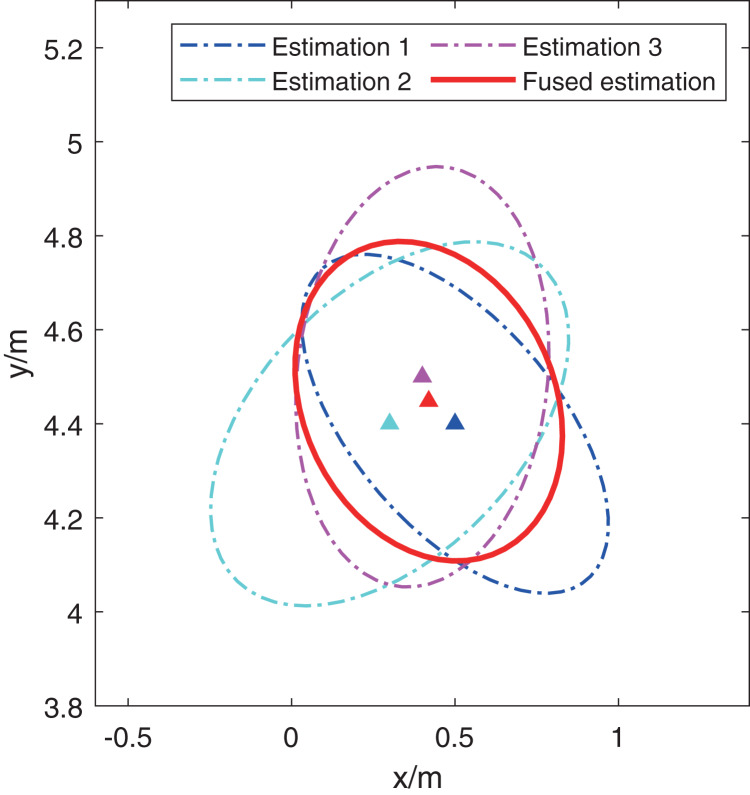
An illustration for fusing three preliminary estimations with the CI method.

Furthermore, this article presents a simple example to illustrate the effectiveness of the CI method, which uses the CI method to fuse three preliminary estimations shown in Fig. 2. In the figure, the red triangle and ellipse denote the fused estimation and covariance respectively. And other three triangles and ellipses with different color represent the three preliminary estimations. It’s obviously that the red ellipse covers the overlap of other three ellipses, which is also the reason that the CI method has a stronger robustness than the classical extended Kalman filter (Li et al., 2020).

### Strategy for eliminating abnormal measurements

Although the CI method can provide a conservative fused estimation, it may not work properly if there are abnormal measurements caused by defective sensors or obstacles between the robots. To handle the problem, the KLD theory can be used to detect the abnormal measurement and eliminate it during the process of measurement update. As we known, the KLD is a method in machine learning to measure the similarity of two probability distributions by quantifying the distance between them (Al Hage, El Najjar & Pomorski, 2017; Wu, Ma & Zhang, 2018).

We assume there are two probability density functions for the same variable $x$, denoted $p(x)$ and $q(x)$ respectively. Then the KL divergence between $p(x)$ and $q(x)$ is defined as

(18) $${D_{KL}}(p||q) = \int_{ - \infty }^{ + \infty } p (x)\log \left( {{{p(x)} \over {q(x)}}} \right)dx$$

For the cooperative localization problem in this article, $p(x)$ and $q(x)$ in Eq. (18) can be viewed as robot’s estimations in the prediction process and the preliminary estimation in the measurements update process, respectively. If robot’s estimations are assumed as multivariate Gaussian distribution, the KL divergence can be represented as

(19) $${D_{KL}}({N_p}||{N_q}) = {1 \over 2}\left[ {{{({\mu _p} - {\mu _q})}^ \top }\Sigma _q^{ - 1}({\mu _p} - {\mu _q}) + (\Sigma _q^{ - 1}{\Sigma _p}) - n + \ln \left( {{{\det ({\Sigma _q})} \over {\det ({\Sigma _p})}}} \right)} \right]$$

where ${\mu _i}$ and ${\Sigma _i}$ are the mean and covariance of the Gaussian distribution respectively, det $( \cdot )$ is the determinant of a matrix, and $n$ is the dimension of robot’s state.

After adding the KLD theory into the cooperative localization process, the whole algorithm can be summarized as Algorithm 2. In the algorithm, lines $1\sim 3$ are the initialization operations including the inputs of robots’ estimation in the prediction stage, the preliminary estimations in the measurements update process, and a threshold $\chi$. The threshold $\chi$ here is to detect the abnormal measurement with the KLD theory, which is chosen as 0.14 by experience in our further simulations. The main operations of the algorithm are presented in lines $4\sim 17$, which are composed by two loops to make all robots complete the localization process. Moreover, the abnormal measurement detection operations with the KLD theory are presented in lines $10\sim 14$.

**Algorithm 2:** Cooperative localization algorithm based on CI method

**Initialization:**
1: Input the state estimations for all robots at time *k*, $\{ (X_k^{{R_i}},P_k^{{R_i}})\}$, $i \in \{ 1,2, \ldots ,n\}$.
2: Input the control signal $u_k^{{R_i}}$ at time *k* and the measurement $Z_{k + 1}^{{R_i},{R_j}}$ at time $k + 1$, where *R*_*j*_ is the robots that existing a communication with *R*_*i*_.
3: Input the threshold *χ* to detect the abnormal measurement.
**Iteration:**
4: **for** each robot *R*_*i*_, $i \in 1, \ldots ,n$ **do**
5: Generate robot *R*_*i*_’s state estimation $\{ (X_{k + 1\mid k}^{{R_i}},P_{k + 1\mid k}^{{R_i}})\}$ according to Eqs. (6) and (8).
6: **for** each measurement $Z_{k + 1}^{{R_i},{R_j}}$ from robot *R*_*j*_ **do**
7: Adjust the measurement noise of $Z_{k + 1}^{{R_i},{R_j}}$ according to Algorithm 1
8: Generate the preliminary estimation $\{ X_{k + 1}^{{R_i},{R_j}},P_k^{{R_i},{R_j}}\}$ according to Eq. (9)
9: Calculate the KLD value *D*_*KL*_ between $\{ (X_k^{{R_i}},P_k^{{R_i}})\}$ and $\{ X_{k + 1}^{{R_i},{R_j}},P_k^{{R_i},{R_j}}\}$ according to Eq. (19)
10: **if** ${D_{KL}} \le \chi$ **then**
11: Send the preliminary estimation $\{ X_{k + 1}^{{R_i},{R_j}},P_k^{{R_i},{R_j}}\}$ to set *B*
12: **else**
13: Discard the measurement $Z_{k + 1}^{{R_i},{R_j}}$ from *R*_*j*_.
14: **end if**
15: Fuse all the preliminary estimations in set *B* according to Eq. (13), and obtain the final estimation $\{ X_{k + 1}^{{R_i}},P_{k + 1}^{{R_i}}\}$.
16: **end for**
17: **end for**
18: **return** $\{ X_{k + 1}^{{R_i},{R_j}},P_k^{{R_i}}\} ,i \in \{ 1,2, \ldots ,n\}$

## Simulations

In this section, the verification processes have been done in two parts. The first part aims to validate the method of considering robots’ uncertainty. In the second part, the performance of the proposed cooperative localization algorithm is validated through two simulations. These simulations are conducted to demonstrate the algorithm’s performance in scenarios with and without abnormal measurement. The performance of the localization algorithms is evaluated based on the trajectory error of robots.

### Validating the method of considering robots’ uncertainty

To validate the effectiveness of Algorithm 1, we have conducted two simple examples involving two robots. And the results is used to compare with that obtained by the extended Kalman filter. In both of the examples given, the robots’ predicted positions are subject to uncertainty, and accurate measurements are used to perform a one-step update using Eq. (9) through Eq. (12). The error between the updated position and its true positions is then used to evaluate the performance of the algorithm.

**Figure 3:**
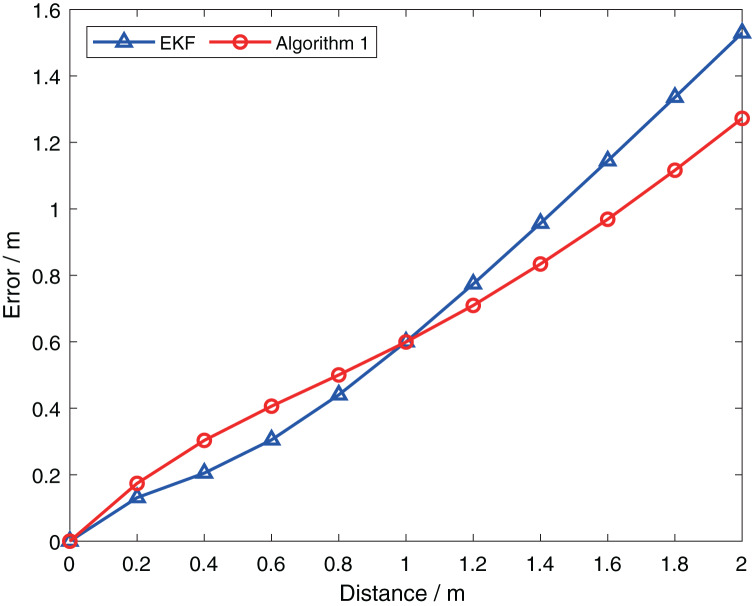
Comparison of performance of two algorithms at different prediction positions.

In the first example, robot $i$ and robot $j$’s true position are at ${[4,0]^ \top }$ and ${[0,0]^ \top }$ respectively. Initially, the predicted positions of both robots, $X_{k + 1|k}^{{R_i}}$ and $X_{k + 1|k}^{{R_j}}$, are at their true positions, with the same covariance $P_{k + 1|k}^{{R_i}} = P_{k + 1|k}^{{R_j}} = diag([0.2,0.2])$. The actual measurement $Z_{k + 1}^{{R_i},{R_j}} = 4$ has measurement noise of ${\eta _{ij}} = 0.1$. We will then move $X_{k + 1|k}^{{R_i}}$ in the positive direction of the x-axis to obtain updated values for different predicted positions, and the results are presented in Fig. 3. For simplicity, the horizontal axis represents the distance between Robot $i$’s predicted position and its true position. Furthermore, the blue line represents the result obtained by the extended Kalman filter, which considers the uncertainty of robot $j$ and requires linearization at both $X_{k + 1|k}^{{R_i}}$ and $X_{k + 1|k}^{{R_j}}$ during the calculation. It is evident that the error obtained by Algorithm 1 is lower than that obtained by the extended Kalman filter when the distance is greater than 1. This indicates that Algorithm 1 accounts for the uncertainty of robot $j$ during the update process, which avoids the large errors caused by two linearizations when using the extended Kalman filter method. When the distance is small, the errors introduced by linearization are small. So the error of Algorithm 1 is slightly greater than that of the extended Kalman filter, but still within the acceptable range.

**Figure 4:**
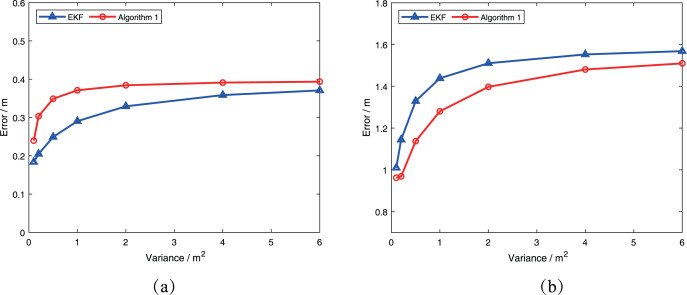
Comparison of performance of two algorithms at different covariance: (A) $X_{k + 1|k}^{{R_i}} = {[4.4,0]^ \top }$; (B) $X_{k + 1|k}^{{R_i}} = {[5.6,0]^ \top }$.

In the second example, we consider two cases where $X_{k + 1|k}^{{R_i}}$ is at ${[4.4,0]^ \top }$ and ${[5.6,0]^ \top }$, respectively. All other parameters are the same as in the first example. We then vary the covariance of robot $j$ to investigate the effect of covariance on Algorithm 1. For simplicity, we assume that the covariance $P_{k + 1|k}^{{R_j}}$ has the same values on the diagonal. The results are presented in Fig. 4. It is evident that as the covariance of robot $j$ increases, the error obtained by Algorithm 1 and the error obtained by the extended Kalman filter with consideration of robot $j$’s uncertainty become increasingly similar. This demonstrates that Algorithm 1 can successfully consider the uncertainty of robot in the measurement update process.

### Simulation for the case with normal measurement

**Figure 5:**
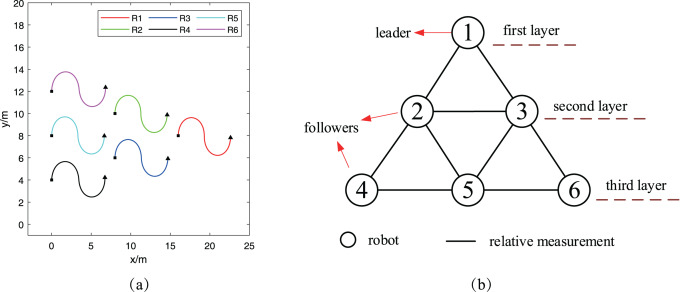
Reference trajectories and communication topology graph: (A) robots’ reference trajectories; (B) communication topology graph.

The first simulation considers the case that six robots ${R_1}\sim {R_6}$ keep a formation to track their reference trajectories shown in Fig. 5A. For each reference trajectory, the square denotes the robot’s start point while the triangle is the terminal point of the robot. The initial states of all robots are presented in Table 1. Corresponding, the variance of all robots’ initial states are set as ${\rm{diag}}([0.2,0.1,0.01])$, except that the variance of ${R_1}$’s initial state is assumed as ${\rm{diag}}([0.01,0.01,0.01])$. Meanwhile, the input noise of all robots are assumed as $Q = {\rm{diag}}([0.2,0.14])$. Furthermore, the communication topology graph between robots is shown in Fig. 5B. It’s easy to find that all the robots can be divided into three layers, while robot ${R_1}$ lies at the first layer and acts as the leader of the formation. And other five robots are the followers and are placed at the other two communication layers.

**Table 1:** Initial states of robots.

ID	Robot ${R_1}$	Robot ${R_2}$	Robot ${R_3}$	Robot ${R_4}$	Robot ${R_5}$	Robot ${R_6}$
Initial state	(16,8, $\pi /2$)	(8,10, $\pi /2$)	(8,6, $\pi /2$)	(0,4, $\pi /2$)	(0,8, $\pi /2$)	(0,12, $\pi /2$)

Obviously, all robots can track their trajectory perfectly if the input noise doesn’t exist. However, the input noise is very common in real scenario, which is considered in this article and assumed as $Q = {\rm{diag}}([0.2,0.14])$ for all robots. Furthermore, among the six robots, robot ${R_1}$ can obtain its position by other localization method while other robots only achieve localization by the relative distance measurements between robots. This assumption is equivalent to the case that there is only one anchor in the environment. According to the principle of two-dimensional localization, there must be at least three anchors if we want to achieve robot positioning, which means robots ${R_2}\sim{R_6}$ in the simulation can’t positioning themselves with the common localization algorithm. In this article, the proposed cooperative localization algorithm can handle this problem and locate other five robots by using the relative measurements. In order to make a comparison, three other methods are also used to locate robots, which are the CI method, the CU method and the method proposed in Wang et al. (2021), respectively. Among those methods, both Wang et al. (2021) and the general CU method use the same way to fuse the local estimations. But Wang et al. (2021) has considered the cross-correlation term of covariance and the general CU method haven’t, which is also the differences between Wang et al. (2021) and the general CU method. Similarly, the main differences between the general CI method and the proposed method in this article are that the proposed method has taken into consideration the uncertainty of robots’ position and has used the KLD to eliminate abnormal measurements, while the general CI method has not.

**Figure 6:**
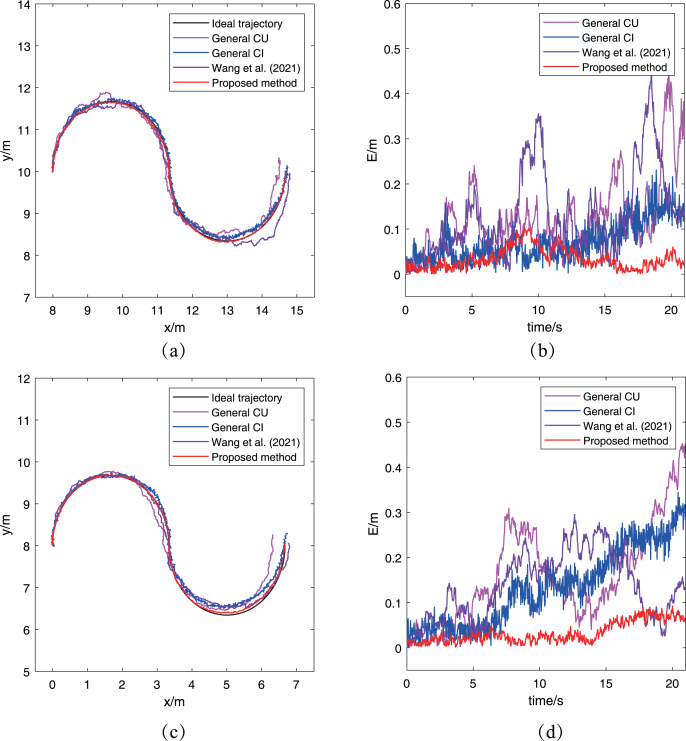
Trajectories of different robots obtained by the proposed algorithm for the case without abnormal measurements: (A) ${R_2}$’s trajectory; (B) ${R_2}$’s trajectory error; (C) ${R_5}$’s trajectory; (D) ${R_5}$’s trajectory error.

For simplicity, each communication layer selects a robot to compare its fused trajectory and trajectory error to evaluate the performance of the proposed algorithm. Figures 6A and 6C have presented ${R_2}$ and ${R_5}$’s fused trajectories and their reference trajectories. It’s easy to find that the fused trajectories obtained by the proposed localization algorithm can match the ideal trajectories for the two robots, while the fused trajectories obtained by other three methods have deviations with the ideal trajectories. In terms of the size of the deviations, the CU method and Wang et al. (2021) have similar performances since their fusion way are same. However, both of them are worse than the performance of the other two methods. For clarity, the trajectory error $E = ||q - {q_d}||$ is also introduced as one evaluation criterion, where $q$ is the estimation of robot’s position, and ${q_d}$ is the corresponding position on the ideal trajectory. And the variation of trajectory error *E* of robots ${R_2}$ and ${R_5}$ with time can be seen in Figs. 6B and 6D respectively. Viewed from the trajectory error changes of robots ${R_2}$ and ${R_5}$, it also be concluded that the performance of the proposed algorithm is the best among the three algorithms, which is same to the conclusion from comparing the robots’ fused trajectories.

Considering that there are multiple robots in this simulation, the root mean square error (RMSE) and the root mean trace error (RMTE) are used to evaluate the overall localization performance for all robots. The two metrics are defined as

(20) $$\matrix{
   {{\rm{RMSE}}} & { = \sqrt {\mathop \sum \limits_{i = 1}^N ||q - {q_d}|{|^2}/N} }  \cr 
   {{\rm{RMTE}}} & { = \sqrt {\mathop \sum \limits_{i = 1}^N {\rm{tr}}({{[\Sigma ]}_i})/N} }  \cr 

 } $$

where *N* is the number of robots and ${\Sigma _i}$ denotes the state covariance of robot ${R_i}$

**Figure 7:**
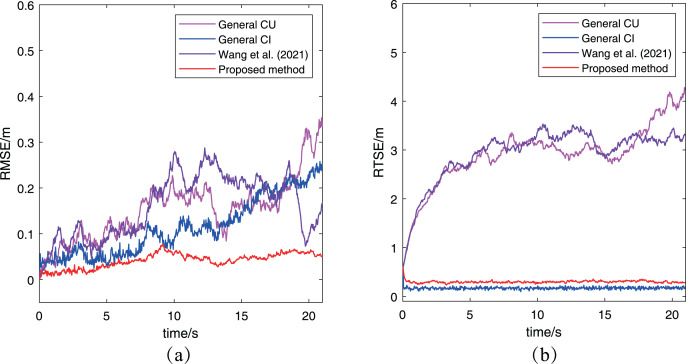
The RMSE and RTSE metrics of localization algorithms for the case without abnormal measurements: (A) RMSE changes with time; (B) RTSE changes with time.

Figure 7 has presented the two metrics change with time. In Fig. 7A, the RMSE of the proposed algorithm is obviously smaller than that of other two methods, which keeps same to the previous conclusion. For the RMTE metric, it’s easy to find that both the proposed algorithm and the general CI method keep a smaller error than the general CU method and Wang et al. (2021). The reason for this phenomenon is that the CU method generally increases the final fused estimation’s covariance to include all estimations’ covariance, while the CI method determines the final covariance by including the intersection part of all estimations’ covariance. Furthermore, the proposed algorithm has a bigger RMTE than the general CI method, which is caused by the proposed algorithm to adopt the approximate method in Algorithm 1 to adjust the measurement noise. In other words, the proposed algorithm has considered the uncertainty of robots, which leads its preliminary estimations have larger covariance than that in the general CI method. Besides, two methods use the same way to fuse preliminary estimations, so it is a normal situation that the proposed algorithm has a bigger RMTE than the general CI method. It’s worth noting that the result of cooperative localization is evaluated comprehensively by both RMSE and RTSE metrics. Although the RTSE of the proposed method is slightly greater than that of the general CI method, but its RMSE is significantly less than the general CI method. So it can also determine the proposed algorithm has a better performance. After analyzing the results of the other three methods based on the two metrics, it can be concluded that the proposed algorithm performs better than both the CU and CI methods.

### Simulation for the case with abnormal measurement

**Figure 8:**
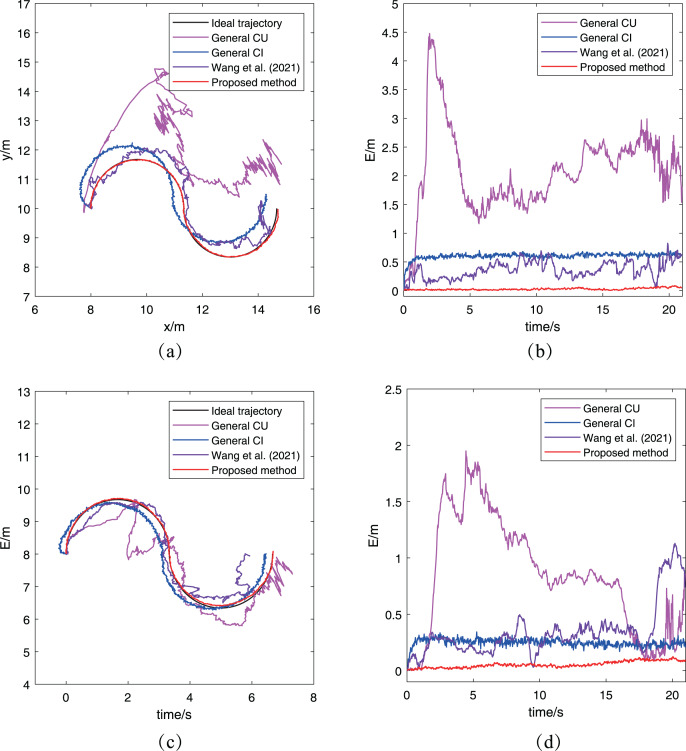
Trajectories of different robots obtained by the proposed algorithm for the case with abnormal measurements: (A) ${R_2}$’s trajectory; (B) ${R_2}$’s trajectory error; (C) ${R_5}$’s trajectory; (D) ${R_5}$’s trajectory error.

The second simulation is conducted to validate the performance of the proposed algorithm when robots have abnormal measurements. In this simulation, an assumption is made that there exists a 1 m distance deviation on the measurement between robots ${R_1}$ and ${R_2}$. Other conditions are same to that in the previous simulation. Similarly, robots ${R_2}$ and ${R_5}$ are chosen to compare their trajectories. Figure 8 has presented the comparison results.

In Fig. 8A, four fused trajectories with using different algorithms are compared for robot ${R_2}$. There is no doubt that the proposed algorithm exhibits the best performance, as its fused trajectory perfectly matches the ideal trajectory. Moreover, the fused trajectory obtained by the CU method is very disorganized after receiving an abnormal measurement, while the CI method can make the fused trajectory similar to the ideal trajectory with relatively stable deviation error. As for the result of the method in Wang et al. (2021), it shows an obvious improvement than the two general method since the cross-correlation term of covariance in Wang et al. (2021) has a small inhibitory effect to the abnormal measurement. And the trajectory error in Fig. 8B shows a similar phenomenon, which illustrates that the proposed algorithm performs well even in cases with abnormal measurements. For the trajectory of robot ${R_5}$, the result of the proposed algorithm also has a better performance compared with the other three algorithms. It’s worth noting that robot ${R_5}$ does not receive any abnormal measurement, but its fused trajectory is still affected by the abnormal measurement between robots ${R_1}$ and ${R_2}$. This is due to the characteristics of cooperative localization, that is, the estimation accuracy of each robot will also affect its neighboring robots’ estimation. It also illustrates the necessity of eliminating the estimation with large deviation during the process of cooperative localization.

**Figure 9:**
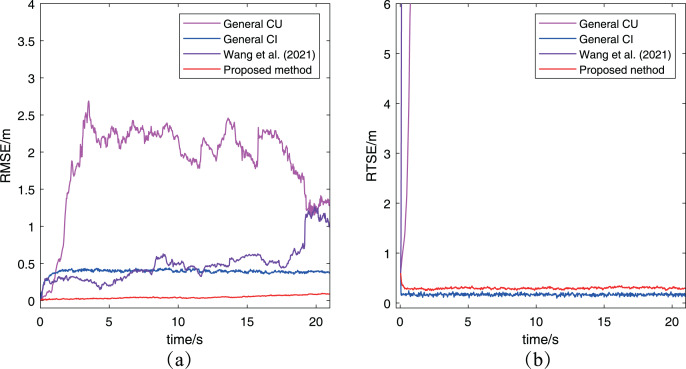
The RMSE and RTSE metrics of localization algorithms for the case with abnormal measurement: (A) RMSE changes with time; (B) RTSE changes with time.

Furthermore, the RMSE and RMTE metrics’ change are also presented in Fig. 9. It’s obvious that the RMSE of the proposed algorithm has been kept at a low level and much better than that of the other three algorithms. And the reason is that the abnormal measurement of robot ${R_2}$ has been eliminated in the process of cooperative localization so as to avoid interfering with other robots. In Fig. 9B, the RMTE of the CU method and Wang et al. (2021) increases rapidly to more than $6$ and never decreases again, while the other two algorithms’ RMTE remain relatively small. However, the performance of the proposed algorithm is the best among the three cooperative localization algorithms after combining the RMSE and RMTE metrics. It also demonstrates only the proposed algorithm has the robustness for the case with abnormal measurements.

**Figure 10:**
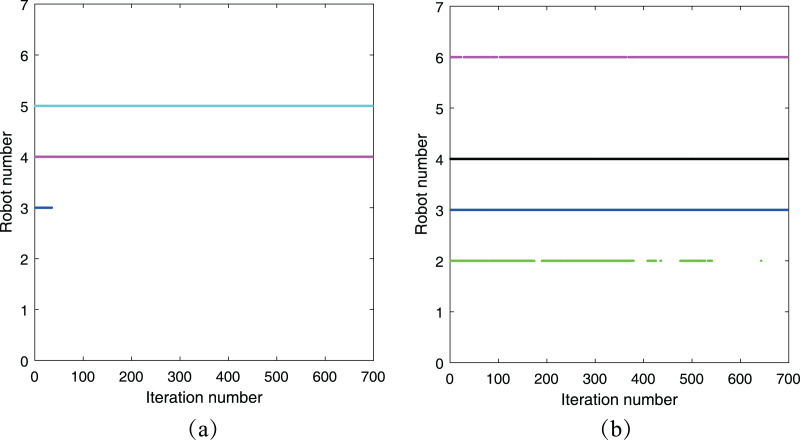
Acceptance of preliminary estimation: (A) ${R_2}$’s neighbor robots; (B) ${R_5}$’s neighbor robots.

To demonstrate the impact of the threshold $\chi$ on eliminating abnormal measurements, we have included a summary of the acceptance of preliminary estimations for both ${R_2}$ and ${R_5}$ in this simulation. This summary is presented in Fig. 10, where the horizontal axis represents the number of iteration steps, and the vertical axis represents the robot number. The colored line consists of a series of points, which indicate that the corresponding robot has accepted the preliminary estimation from its neighboring robots. From Fig. 10A, it is evident that robot ${R_2}$ did not accept the preliminary estimation from robot ${R_1}$ throughout the simulation period due to the presence of abnormal measurements between them. Moreover, robot ${R_1}$ accepted the preliminary estimation from robot ${R_3}$ initially but later rejected it when the distance between their estimations exceeded the threshold $\chi$. A similar phenomenon is observed in Fig. 10B, highlighting the effectiveness of the threshold $\chi$ in the cooperative localization process.

## Conclusions

In this article, the cooperative localization problem is studied and a novel localization algorithm based on CI method is proposed to achieve accurate localization for multi-robot system. The proposed cooperative localization algorithm can locate robots by using the relative distance measurement with few anchors in the environment. In order to improve the accuracy of algorithm, an approximation method based on particles are proposed to adjust the measurement noise with considering the uncertainty of robots’ position. Furthermore, the KLD theory is used to detect the abnormal measurement and improve the robustness of the localization algorithm. Finally, two simulations are conducted to validate the performance of the proposed algorithm. The results indicate that that the proposed algorithm can attain a high degree of localization accuracy for robots and exhibit robustness in scenarios involving abnormal measurements.

## Supplemental Information

10.7717/peerj-cs.1373/supp-1 Supplemental Information 1 Raw data for Figures 3, 4, 6, 7, 8 and 9.

10.7717/peerj-cs.1373/supp-2 Supplemental Information 2 All codes for the simulations and figures. The “figure” file folder contains the codes for plotting the illustration of covariance intersection. The “path” file folder contains the codes for plotting all simulation figures.

## Additional Information and Declarations

### Data Availability

The following information was supplied regarding data availability: The code is available in the Supplemental File.
